# Tonabersat suppresses priming/activation of the NOD-like receptor protein-3 (NLRP3) inflammasome and decreases renal tubular epithelial-to-macrophage crosstalk in a model of diabetic kidney disease

**DOI:** 10.1186/s12964-024-01728-1

**Published:** 2024-07-05

**Authors:** C. L. Cliff, P. E. Squires, C. E. Hills

**Affiliations:** Joseph Banks Laboratories, School of Life and Environmental Sciences, University of Lincoln, Lincoln, LN6 7DL UK

**Keywords:** Tonabersat, Hyperglycaemia, NLRP3, Inflammasome, Inflammation, Diabetic nephropathy, Connexin-43, Hemichannel, Cell-cell communication, Macrophages

## Abstract

**Background:**

Accompanied by activation of the NOD-like receptor protein 3 (NLRP3) inflammasome, aberrant connexin 43 (Cx43) hemichannel-mediated ATP release is situated upstream of inflammasome assembly and inflammation and contributes to multiple secondary complications of diabetes and associated cardiometabolic comorbidities. Evidence suggests there may be a link between Cx43 hemichannel activity and inflammation in the diabetic kidney. The consequences of blocking tubular Cx43 hemichannel-mediated ATP release in priming/activation of the NLRP3 inflammasome in a model of diabetic kidney disease (DKD) was investigated. We examined downstream markers of inflammation and the proinflammatory and chemoattractant role of the tubular secretome on macrophage recruitment and activation.

**Methods:**

Analysis of human transcriptomic data from the Nephroseq repository correlated gene expression to renal function in DKD. Primary human renal proximal tubule epithelial cells (RPTECs) and monocyte-derived macrophages (MDMs) were cultured in high glucose and inflammatory cytokines as a model of DKD to assess Cx43 hemichannel activity, NLRP3 inflammasome activation and epithelial-to-macrophage paracrine-mediated crosstalk. Tonabersat assessed a role for Cx43 hemichannels.

**Results:**

Transcriptomic analysis from renal biopsies of patients with DKD showed that increased Cx43 and NLRP3 expression correlated with declining glomerular filtration rate (GFR) and increased proteinuria. In vitro, Tonabersat blocked glucose/cytokine-dependant increases in Cx43 hemichannel-mediated ATP release and reduced expression of inflammatory markers and NLRP3 inflammasome activation in RPTECs. We observed a reciprocal relationship in which NLRP3 activity exacerbated increased Cx43 expression and hemichannel-mediated ATP release, events driven by nuclear factor kappa-B (NFκB)-mediated priming and Cx43 hemichannel opening, changes blocked by Tonabersat. Conditioned media (CM) from RPTECs treated with high glucose/cytokines increased expression of inflammatory markers in MDMs, an effect reduced when macrophages were pre-treated with Tonabersat. Co-culture using conditioned media from Tonabersat-treated RPTECs dampened macrophage inflammatory marker expression and reduced macrophage migration.

**Conclusion:**

Using a model of DKD, we report for the first time that high glucose and inflammatory cytokines trigger aberrant Cx43 hemichannel activity, events that instigate NLRP3-induced inflammation in RPTECs and epithelial-to-macrophage crosstalk. Recapitulating observations previously reported in diabetic retinopathy, these data suggest that Cx43 hemichannel blockers (i.e., Tonabersat) may dampen multi-system damage observed in secondary complications of diabetes.

## Background

Diabetic nephropathy (DN) is the leading cause of end-stage renal failure [1] and a major microvascular complication of diabetes associated with an increased risk of cardiovascular disease [2–4] and diabetic retinopathy [5–7]. In the absence of curative therapies, current treatment is informed by stage and aetiology of disease. Centred around the regulation of blood glucose, lipids, and blood pressure, a four-pillared approach is recommended, which includes renin-angiotensin system (RAS) blockers, non-steroidal mineralocorticoid receptor antagonists, sodium-glucose co-transporter-2 (SGLT2) inhibitors and glucagon-like peptide 1 (GLP-1) receptor agonists [8, 9]. Whilst these drugs improve cardiorenal outcomes, for many, kidney failure remains inevitable. Consequently, further work identifying new therapeutic approaches that can target late-stage inflammatory damage and slow entry into end-stage renal disease (ESRD) are urgently needed.

Contributed to by multiple interrelated pathophysiological mechanisms involving haemodynamic, metabolic, and inflammatory pathways, tubulointerstitial inflammation and fibrosis of the renal proximal tubules dictates disease severity and progression [10–12]. Moreover, inflammation accounts for residual risk across multiple secondary complications and associated cardiometabolic comorbidities [13–17]. Integral to this inflammatory damage is the NOD-like receptor protein 3 (NLRP3) inflammasome, a major immune complex which regulates the innate inflammatory response to both endogenous (e.g., adenosine triphosphate [ATP]) and exogenous (e.g., pathogens) stimuli [18]. Linked to numerous age-associated conditions of chronic inflammation [19–22] and multiple complications of diabetes [23–27], an intervention which selectively blocks NLRP3 inflammasome activation in a sterile setting would undoubtedly maximise positive patient outcomes and potentially lessen reliance on polypharmacy [28].

Patients with diabetic nephropathy are at higher risk of developing diabetic retinopathy [7, 29]. Inflammation in retinopathy has been widely linked to the NLRP3 inflammasome [30], with recent studies suggesting that aberrant connexin 43 (Cx43) hemichannel activity sits upstream of its assembly [31, 32]. Connexins are transmembrane proteins that oligomerise into small pores termed ‘hemichannels’ [33]. Normally closed under physiological conditions, they open in disease to release small ions and molecules into the intercellular space. One such molecule is ATP, a danger-associated molecular pattern (DAMP) and recognised stimulus of NLRP3 complex assembly and activation [34]. A two-step process comprised of nuclear factor kappa-B (NFκB)-mediated interleukin-(IL)1β priming (step 1) and ATP-P2 × 7 purinergic receptor activation (step 2), formation of the NLRP3 inflammasome complex [35] culminates in caspase 1-mediated cleavage of pro-IL1β and pro-IL18 [36]. Downstream activation of pro-inflammatory cytokines leads to inflammatory cell death (pyroptosis) and immune cell activation. Retinal pigment epithelial (RPE) cells treated with high glucose and a combination of inflammatory cytokines exhibit increased NLRP3 complex formation and trigger release of pro-inflammatory cytokines IL6, IL8, monocyte chemoattractant protein-1 (MCP1), intercellular cell adhesion molecule-1 (ICAM-1) and vascular endothelial growth factor (VEGF) [37]. Blocking Cx43 hemichannel-ATP release in RPE cells using Peptide 5 prevented the upregulation of inflammasome formation and cytokine release, a response blunted by the exogenous addition of ATP [37]. Similarly, in both an in vivo [32] and human retinal explant model of retinopathy [38], Cx43 hemichannel blockers Peptide 5 and Tonabersat prevented inflammasome complex formation. Cumulatively, inhibition of Cx43 hemichannels and resultant impaired inflammasome assembly reduced vascular leak and oedema, thereby protecting against severity of damage in the diabetic eye [32, 38].

Many of the major mechanisms that contribute to kidney damage e.g., inflammation and oxidative stress, are common to the underlying pathology observed at the back of the eye in disease [39–41]. Given the link between Cx43 and the NLRP3 inflammasome in retinopathy [42], we recently explored the relationship between these two proteins in chronic kidney disease. We identified that increased Cx43 expression in biopsy material isolated from individuals with kidney disease, correlated positively with declining GFR and increased proteinuria. Moreover, using a unilateral ureteral obstruction (UUO) in vivo mouse model of advanced kidney damage, we determined that a reduction in Cx43 expression (tubule-directed Cx43^−/−^ UUO) or pharmacological inhibition (Peptide 5) of Cx43 hemichannels protects the kidneys from injury and paralleled a significant reduction in gene expression of NLRP3, IL1ß and gene expression of associated downstream pro-inflammatory mediators. Importantly, Peptide 5 blocked the UUO-induced increase in macrophage infiltration and chemoattractant expression (e.g., MCP1), effects recapitulated in our tubule-directed Cx43^−/−^ UUO mice [43]. As the primary immune cell in the kidney, macrophage accumulation is a prognostic factor for DN [44], with cells preferentially polarising into an M1 pro-inflammatory phenotype [45] and infiltration directly proportional to severity of injury [46–48]. In support of our in vivo observations, co-culture experiments demonstrate M2-to-M1 macrophage polarisation induced by tubule cell-derived factors [49], whilst a recent study by Xu et al. reported increased macrophage pyroptosis when bone marrow-derived macrophages were co-cultured with RPTECs isolated from the tubule-directed Cx43^−/−^ UUO mouse [50]. Macrophage infiltration is reduced in the tubule-directed Cx43^−/−^ UUO mouse with gene expression of NLRP3 and IL1ß diminished in both Cx43^−/−^ tubule cells [43] and in Cx43 small interfering RNA (siRNA) treated macrophages [51] However, no studies have yet explored the potential role and downstream effects for Cx43 hemichannel activity in paracrine-mediated communication between kidney epithelial and immune cells under conditions of high glucose and inflammation.

Tonabersat is a hemichannel blocker currently in phase 2b trials for diabetic macular oedema (NCT05727891). It has previously been shown to successfully reduce NLRP3-induced inflammation in the retinal pigment epithelium through suppression of Cx43 hemichannel activity. Its effects on behaviour on different cell types in the diabetic kidney and NLRP3 inflammasome signalling are yet to be elucidated.

In the current study, we cultured primary human renal proximal tubule epithelial cells (RPTECs) and healthy donor monocyte-derived macrophages (MDMs) in a combination of high glucose and inflammatory cytokines, to generate an in vitro model of DN. We investigated a role for Cx43 hemichannel activity in tubule cell NLRP3 inflammasome activation and downstream markers of inflammation ahead of evaluating the implications of blocking these events on paracrine-mediated cell crosstalk. We demonstrate for the first time that tubular Cx43 hemichannel-mediated ATP release triggers a series of downstream events which primes and activates the NLRP3 inflammasome in response to high glucose, events which are blocked in cells pre-incubated with Tonabersat. Moreover, Tonabersat blocks the increased expression of downstream markers of inflammation, whilst lessening the pro-inflammatory and chemoattractant nature of the cellular secretome. Indirect and direct co-culture of MDMs with conditioned media from treated RPTECs demonstrates increased macrophage migration, expression of inflammatory markers, and NLRP3 inflammasome activation. Importantly, several of these events were significantly lessened when Cx43 hemichannel activity was blocked in either tubule cells or macrophages, suggesting that Cx43 hemichannels play a central role in orchestrating both NLRP3 inflammasome-induced inflammation and paracrine-mediated cell communication in the diabetic kidney.

## Methods

### Culture of human primary renal proximal tubule epithelial cells

Primary human RPTECs isolated from a normal, healthy kidney were purchased from American Type Culture Collection (ATCC, Manassas, US) and Lonza (Basel, Switzerland). Cells were cultured (up to 3 passages) in Renal Epithelial Cell Basal Medium supplemented with the Renal Epithelial Cell Growth Kit (PCS-400-040) and Penicillin-Streptomycin-Amphotericin B Solution (ATCC, Manassas, US) at 37 °C in a humidified environment with 5% CO_2_. Media was replaced every 2 days and cells were passaged when they reached 90–95% confluency. Cells were seeded at 5,000 cells per cm^2^ and cultured to 85–90% confluency. RPTECs were treated with growth media adjusted to contain basal (5mmol/L) or high (25mmol/L) glucose ± IL1β (10ng/mL; Miltenyi Biotec, Germany) and tumour necrosis factor alpha (TNFα; 10ng/mL; Miltenyi Biotec, Germany). Cells were also pre-treated for 30 min with inhibitors Tonabersat (50µM; Sigma Aldrich, US), YVAD CMK (10–100µM; Bio-Techne, UK), BAY11 7082 (5µM; Sigma Aldrich, US), PD98059 (50µM; Tocris Bioscience, UK), SB203580 (10µM; Tocris Bioscience, UK) and 1,2-bis(o-aminophenoxy)ethane-N, N,N′,N′-tetraacetic acid (BAPTA-AM) (5µM; Abcam, UK).

### Isolation of primary peripheral blood mononuclear cells

Blood samples (~ 16 mL) were taken with informed consent (University of Lincoln LEASNo: UoL2022_8714) from eight healthy human volunteers. Peripheral blood mononuclear cells (PBMCs) were isolated using density gradient centrifugation. Blood was layered on top of Ficoll Paque Plus media (Fisher Scientific, US) at a 3:1 ratio and centrifuged at 400 relative centrifugal force (RCF) for 40 min with the brakes off. PBMCs were isolated from the interphase and washed (x2) in 10mL phosphate buffered saline (PBS), ahead of a 10 min centrifuge spin at 600 RCF for 10 min with the brakes on. Isolated PBMCs were then resuspended and cultured in Gibco RPMI-1640 media (Fisher Scientific, US) with 10% foetal calf serum (FCS).

### Differentiation of human primary monocytes to macrophages

Primary human monocytes were isolated from PBMCs using cluster of differentiation-(CD)14 microbeads (Miltenyi Biotec, Germany) following manufacturer’s instructions. Monocytes were seeded at 8 × 10^5^ cells/mL in RPMI culture media with 10% FCS. After a 2 h rest period cells were treated with macrophage colony stimulating factor (M-CSF; 20ng/mL; Miltenyi Biotec, Germany) to induce differentiation of monocytes to macrophages. After 3- and 5-days media was replaced with M-CSF (10ng/ml) containing RPMI media with 10% FCS. After 7 days incubation with M-CSF, cells were treated as required for experiments.

### Co-culture

For co-culture experiments, healthy human MDMs were cultured in cell supernatant from treated RPTECs for 2 h. Where appropriate, cells were pre-treated for 30 min with Tonabersat (50µM) prior to and during incubation with RPTEC media.

### Monocyte recruitment and macrophage migration assay

Primary human monocytes were seeded and differentiated in transwell inserts (5µM; Sarstedt, Germany). Before and following differentiation, transwells were transferred to wells containing cell supernatant from treated RPTECs for 2 h. Transwells were removed and cells within the wells were trypsinised and counted as a measure of cell recruitment/migration.

### Transcriptomic analysis

Data from the Nephroseq (www.nephroseq.org, University of Michigan, Ann Arbor, MI, USA) database were extracted to examine changes in gene expression in healthy human kidneys compared with kidneys from people with DN. Expression data was obtained from the ‘Woroniecka Diabetes TubInt’ [52] dataset consisting of a total of 12 healthy human donors and 10 DN patients’ micro-dissected human kidney tubulointerstitial samples. Expression data was also obtained from the ‘Schmid Diabetes TubInt (22)’ [53] dataset in which 11 renal tubulointerstitial human biopsy samples were analysed and the ‘Ju CKD TubInt’ [54] comprised of 170 micro-dissected tubulointerstitial samples from individuals with chronic kidney disease (CKD) and 31 healthy living donors. Samples in all datasets were analysed using an Affymetrix human genome U133A 2.0 microarray. This dataset was previously named Schmid Diabetes.

### Carboxyfluorescein dye uptake

Primary RPTECs were cultured on fluorodishes (23.5 mm diameter; World Precision Instruments, Hertfordshire, UK). Following treatment, cells were incubated with Ca^2+^-free (+ Ethylene glycol-bis(β-aminoethyl ether)-N, N,N′,N′-tetraacetic acid [EGTA]; 1mM) balanced salt solution (BSS; pH 7), composed of NaCl (137mM), KCl (5.4mM), MgSO_4_ (0.8mM), Na_2_HPO_4_ (0.3mM), KH_2_PO_4_ (0.4mM), NaHCO_3_ (4.2mM), 4-(2-hydroxyethyl)-1-piperazineethanesulfonic acid (HEPES; 10mM) and glucose (5mM), with 5,6-carboxyfluorescein (200µM; Fisher Scientific, US) for 10 min, allowing uptake of the fluorescent dye through hemichannels forced open by the artificial (experimental) removal of extracellular Ca^2+^. Calcium-containing (CaCl_2_; 1.3mM) BSS with carboxyfluorescein was added to cells for 5 min to close the hemichannels whilst preventing wash out of dye within the cells. A subsequent wash in Ca^2+^-containing BSS (20mL) maintained the hemichannels in the closed state and removed excess dye from the cell exterior. Images of cells were captured using a Cool Snap HQ charged-coupled device camera (Roper Scientific) and Metamorph software (Universal Imaging Corp., Marlow, Bucks, UK). ImageJ was used to quantify dye uptake by drawing a region of interest around each cell (10–15 cells/dish) and measuring the mean pixel intensity, with background fluorescence subtracted [55].

### ATP-lite luminescence assay

The ATP-lite luminescence assay system [56] (Perkin Elmer, US) was used to measure extracellular ATP following manufacturer’s instructions. Primary human RPTECs were cultured in solid, white 96-well plates in a total volume of 100µL of media, with a 30-minute pre-treatment of anti-ectonucleotidase ARL 67,156 trisodium salt (300µM; R&D Systems, US). Luminescence was detected using a SpectraMax iD3 plate reader (Molecular Devices, US).

### Real-time quantitative polymerase chain reaction (RT-qPCR)

Total RNA was extracted using TRIzol reagent (Invitrogen, US), chloroform and isopropanol following the manufacturer’s instructions. RNA concentration and quality was determined by control of optical density at 260 and 280 nm using a Nanodrop. Cellular RNA (500ng) was converted to single-stranded complementary (c)DNA using a high-capacity cDNA reverse transcription kit (Applied biosystems, US) following the manufacturer’s instructions. Samples were placed into a BioRad T100 Thermo Cycler (BioRad, US) following a three-step protocol; (1) primer annealing- 25 °C for 10 min, (2) DNA polymerisation- 37 °C for 2 h, (3) enzyme denaturation- 85 °C for 5 min. Real-time quantitative (RTq) PCR was performed using qPCRBIO SyGreen Blue Mix, ROX dye (PCR Biosystems, UK) and DNA oligo primers (Table 1). Using a Stepone Plus Real-Time PCR instrument (Applied Biosystems, US) cDNA was denatured at 95 °C for 2 min, followed by 40 amplification cycles of 60 °C for 20 s and 95 °C for 5 s. A standard curve was used to give relative cDNA concentrations which were normalised against expression of housekeeping gene Glyceraldehyde-3-phosphate dehydrogenase (GAPDH). A melt curve analysis of 95 °C for 15 s, 60 °C for 1 min, temperature ramping by 1 °C over 20 min and 95 °C for 15 s confirmed primer specificity and detected possible contamination. Results were analysed using StepOne software v2.3 (Applied Biosystems, US).

**Table 1:** List of human forward and reverse primers used for RT-qPCR.

Target Gene	Forward Primer	Reverse Primer
GAPDH	TTCACCACCATGGAGAAGGC	AGGAGGCATTGCTGATGATCT
Cx43	ATGGGTGACTGGAGCGCCTTAG	CTAGATCTCCAGGTCATCAGG
IL1β	TGGCAGAAGTACCTGAGCTCGC	GCCGCCATCCAGAGGGCAGA
NLRP3	ggactgaagcacctgttgtgca	tcctgagtctcccaaggcattc
IL6	CCTGAGAAAGGAGACATGTAACAAGA	GGAAGGTTCAGGTTGTTTTCTGC
MCP1	GCTCGCTCAGCCAGATGCAA	TCCTGAACCCACTTCTGCTTG
G-CSF	AAGCTGTGCCACCCCGAGGA	GTGGGACCCAACTCGGGGGA
TNFα	GTGATCGGCCCCCAGAGGGAA	TGGAGCTGCCCCTCAGCTTGA
IL1α	CCAGCCAGAGAGGGAGTCATT	CATGGAGTGGGCCATAGCTT
IL10	GGGCACCCAGTCTGAGAACA	GACAAGGCTTGGCAACCCAG
CD206	GCCCGGAGTCAGATCACACA	AGTGGCTCAACCCGATATGACAG
STAT1	TGTATGCCATCCTCGAGAGC	AGACATCCTGCCACCTTGTG
STAT6	CAAAGCCCTAGTGCTGAAGAG	CTCCTGCTGTAGCTGGGAATA
CXCL12	TCAGCCTGAGCTACAGATGC	CTTTAGCTTCGGGTCAATGC
CX3CL1	GGATGCAGCCTCACAGTCCTTAC	GGCCTCAGGGTCCAAAGACA

### Western blotting

Protein from whole cell lysate was isolated, separated by SDS-PAGE gel electrophoresis and transferred onto Immobilon-FL PVDF membranes as previously described [57]. Membranes were blocked with Odyssey blocking buffer (LI-COR, US), prior to probing for Cx43 (1:1000). Bands were visualised using secondary antibodies (Goat anti-Mouse IgG 680, Goat anti-Rabbit IgG 800) and imaged using a Licor Odyssey FC and semi-quantified using Licor Image Studio (v5.2, LI-COR, US). Values were normalised against anti-α-tubulin (1:40,000).

### Caspase Glo-1 inflammasome assay

Caspase 1 activity was quantified using the Caspase-Glo-1 Inflammasome Assay (Promega, US) following manufacturer’s instructions. Primary human RPTECs were cultured in solid, white 96-well plates in a total volume of 50µL media. Luminescence was detected using a SpectraMax iD3 plate reader (Molecular Devices, US). Caspase inhibitor AC-YVAD-CHO (1µM) was used as a negative control.

### IL1β enzyme linked immunosorbent assay (ELISA)

Secretion of active human IL1β into cell supernatant was assessed using a human IL1β/IL1F2 quantikine enzyme linked immunosorbent assay (ELISA; R&D Systems, US) following the manufacturer’s instructions. The optical density at 540 nm with wavelength correction was measured using a SpectraMax iD3 plate reader (Molecular Devices, US). Standard values were used to create a line of best fit.

### Statistical analysis

Data are presented as mean ± standard error of the mean (SEM) unless otherwise stated. Statistical analysis of data was performed using a one-way analysis of variance (ANOVA) with Tukey’s multiple comparison post-test, T-test with Welch’s correction analysis or simple linear regression. Analysis was performed using GraphPad Prism v9.4 (GraphPad Software, USA). Values of *P* < 0.05 were considered statistically significant.

## Results

### Expression of Cx43 is elevated in diabetic nephropathy and correlates with declining renal function

**Fig. 1:**
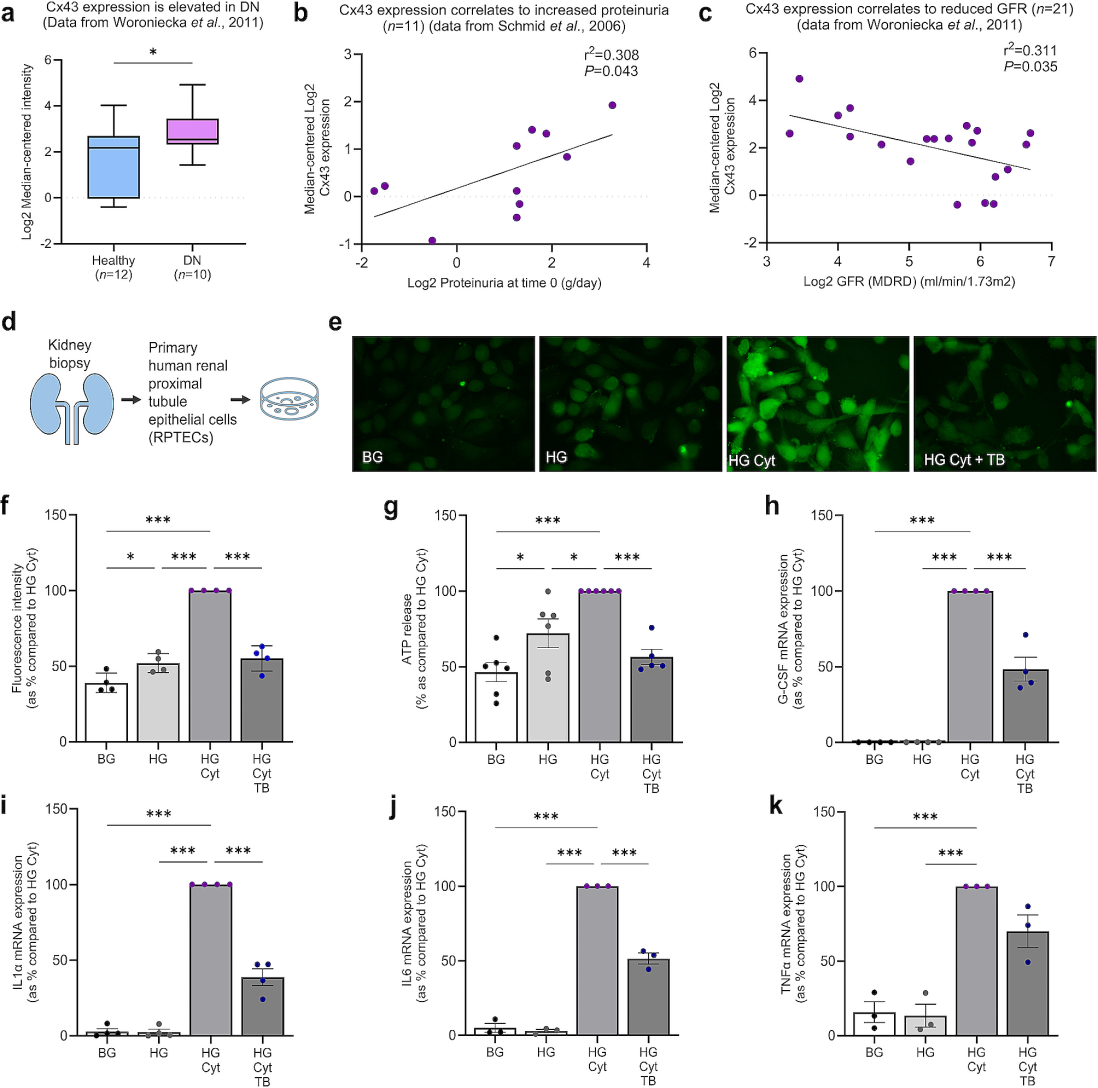
Tonabersat blocks high glucose and pro-inflammatory cytokine-mediated increases in Cx43 hemichannel activity and expression of pro-inflammatory markers in primary human RPTECs. Analysis of Nephroseq transcriptomic datasets show that **(a)***GJA1* (Cx43) expression increases in kidneys from patients with DN (*n* = 10 with DN and *n* = 12 healthy controls) [52] which positively correlates to **(b)** proteinuria [53] and **(c)** declining GFR [52]. Primary RPTECs **(d)** were treated with 5mM/L basal glucose (BG) or 25mM/L high glucose (HG) +/- IL1β (10ng/mL) and TNFα (10ng/mL; Cyt) +/- Tonabersat (TB; 50µM) for 48 h. Carboxyfluorescein dye uptake studies **(e)** determined change in hemichannel number at the cell surface which was quantified using Fiji software **(f)**. An ATPlite luminescence assay measured cellular release of ATP into the supernatant **(g)**. Use of RT-qPCR evaluated changes in mRNA expression of G-CSF **(h)**, IL1α **(i)**, IL6 **(j)** and TNFα **(k)**, normalised against GAPDH. All groups represent *n* = 3–6 unless otherwise specified. ANOVA and Tukey post-test were used for experimental comparisons except for transcriptomic data where an unpaired t-test with Welch’s correction and simple linear regression were used for statistical analysis. Significance is displayed as *P < 0.05 and ***P < 0.001

Whilst studies in experimental models of kidney injury demonstrate a link between increased Cx43 expression and inflammation [16, 50, 58], Cx43 expression in human DN remains to be reported. Using human datasets of renal transcriptomic data available on the Nephroseq repository (Fig. 1a-c) [52–54], we assessed the expression of Cx43 in healthy (*n* = 12) and diseased (*n* = 10) kidneys and determined a significant increase in Cx43 (*GJA1*) mRNA expression in biopsy samples isolated from people with DN as opposed to healthy donor controls (*P <* 0.05; Fig. 1a). Subsequent analysis determined that increased Cx43 expression in disease positively correlates with increasing proteinuria (*P <* 0.05; Fig. 1b) and declining GFR (*P <* 0.05; Fig. 1c).

### Tonabersat blocks high glucose-induced hemichannel-mediated ATP release and expression of inflammatory cytokines in primary human RPTECs

Having ascertained that Cx43 expression is increased in biopsy material from people with diabetes and kidney disease, and that this expression correlates with declining renal function, we utilised an in vitro model of DKD (Fig. 1d) to evaluate the role of Cx43 hemichannels in regulating the expression of inflammatory cytokines and the efficacy of Tonabersat in blocking this response. Carboxyfluorescein dye uptake studies determined the extent of hemichannel opening in human RPTECs (Fig. 1e) treated with high glucose +/- a combination of inflammatory cytokines IL1β and TNFα. Dye uptake in high glucose and cytokine treated cells increased by 61% (*P <* 0.001; Fig. 1e&f) as compared with basal glucose alone, a change paralleled by a 54% increase in ATP release (*P <* 0.001; Fig. 1g). Pre-incubation with Tonabersat blunted the high glucose and cytokine-induced increase in both dye uptake (45 ± 4.2%; *P <* 0.001) and ATP release (44 ± 5.0%; *P <* 0.001).

Knock-down of Cx43 in the heterozygous Cx43^+/−^ UUO mouse and deletion of Cx43 in the tubule-specific homozygous Cx43^−/−^ UUO mouse protects against inflammation [43, 58]. Here we demonstrate for the first time that Tonabersat is able to block Cx43 hemichannels in human primary RPTECs to significantly dampen a high glucose and cytokine-induced increase in mRNA expression of inflammatory markers granulocyte-colony stimulating factor (G-CSF; 100%; *P <* 0.001; Fig. 1h), IL1α (97.1%; *P <* 0.001; Fig. 1i) and IL6 (95.1%; *P <* 0.001; Fig. 1j) by 52 ± 7.9% (*P <* 0.001) 61 ± 5.5% (*P <* 0.001) and 49 ± 3.7% (*P <* 0.001), respectively. Tonabersat did not significantly reduce a high glucose and cytokine-mediated increase in TNFα (Fig. 1k).

### NLRP3 inflammasome activation in diabetic nephropathy correlates with a decline in renal function

**Fig. 2:**
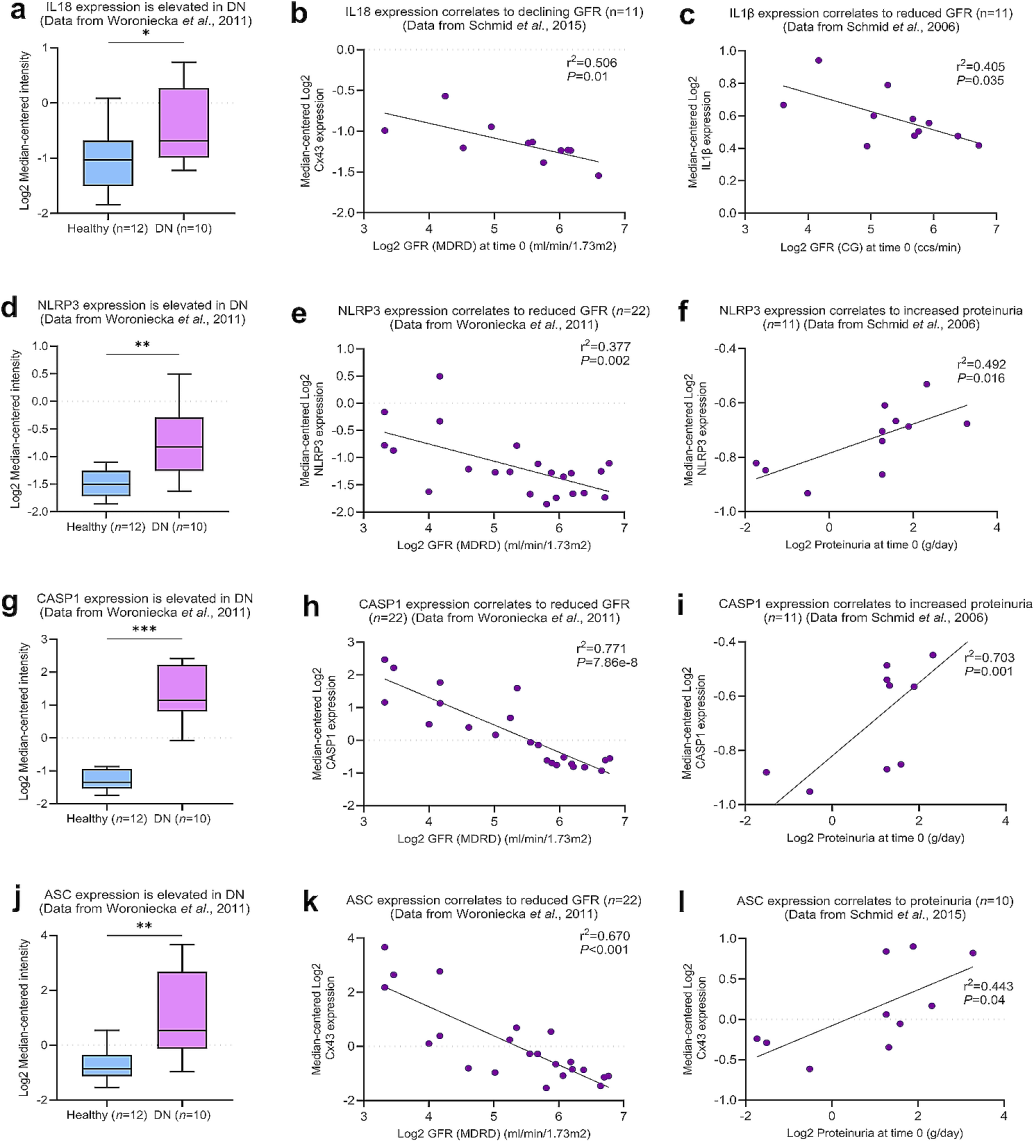
Inflammasome-related gene expression in individuals with diabetic nephropathy correlates to reduced renal function. Transcriptomic analysis was performed on Nephroseq data [52, 53] comparing mRNA expression of IL18 **(a & b)**, IL1β **(c)**, NLRP3 **(d-f)**, caspase 1 (CASP1; **g-i)** and ASC **(j-l)** in kidney biopsies from healthy donors and donors with diabetic nephropathy (DN) and compared with functional parameters. The sample number (*n*) is specified where appropriate. An unpaired t-test with Welch’s correction analysis and simple linear regression were used for statistical analysis. Significance is displayed as *P < 0.05, **P < 0.01, ***P < 0.001

In models of diabetic retinopathy [37, 59, 60] and across other age-associated morbidities [51, 61–63], increased Cx43 hemichannel activity has been shown to contribute to sterile chronic inflammation via activation of the NLRP3 inflammasome. Here we used publicly available transcriptomic datasets and assessed expression of NLRP3 inflammasome components in kidney biopsy samples isolated from individuals with DN. Analysis of data from Woroniecka et al., 2011 [52] determined an increase in IL18 (*P <* 0.05; Fig. 2a), NLRP3 (*P <* 0.01; Fig. 2d), caspase 1 (*P <* 0.001; Fig. 2g) and apoptosis-associated speck-like protein containing a C-terminal caspase recruitment domain (ASC; *P <* 0.01; Fig. 2j) expression in individuals with DN as compared with healthy controls. Moreover, increased mRNA expression of IL18 (Fig. 2b) and IL1β (Fig. 2c), components upregulated during inflammasome priming (step 1), correlate positively with a declining GFR (*P <* 0.01 and *P <* 0.05 respectively). Linked to reduced renal function, increased expression of NLRP3, caspase 1 (CASP1), and ASC positively correlate with a declining GFR (*P <* 0.01, *P <* 0.001, *P <* 0.05; Fig. 2e, 2h and 2k respectively) and increased proteinuria (*P <* 0.05, *P <* 0.001, *P <* 0.001; Fig. 2f, i and l respectively) in DN.

### Tonabersat blocks hemichannel-mediated priming and activation of the NLRP3 inflammasome in RPTECs

Data in Fig. 1 provides novel evidence that Tonabersat successfully blocks Cx43 hemichannel-mediated changes in pro-inflammatory cytokine gene expression in RPTECs under conditions of high glucose and cytokines. Given the link between Cx43-NLRP3 activation in retinopathy and that increased inflammasome expression in DN correlates with increased proteinuria and declining GFR (Fig. 2), we investigated a role for Cx43 hemichannels in regulating NLRP3 inflammasome activation in primary human RPTECs, when pre-incubated with Tonabersat and cultured in high (25mM) glucose with a cocktail of inflammatory cytokines.

**Fig. 3:**
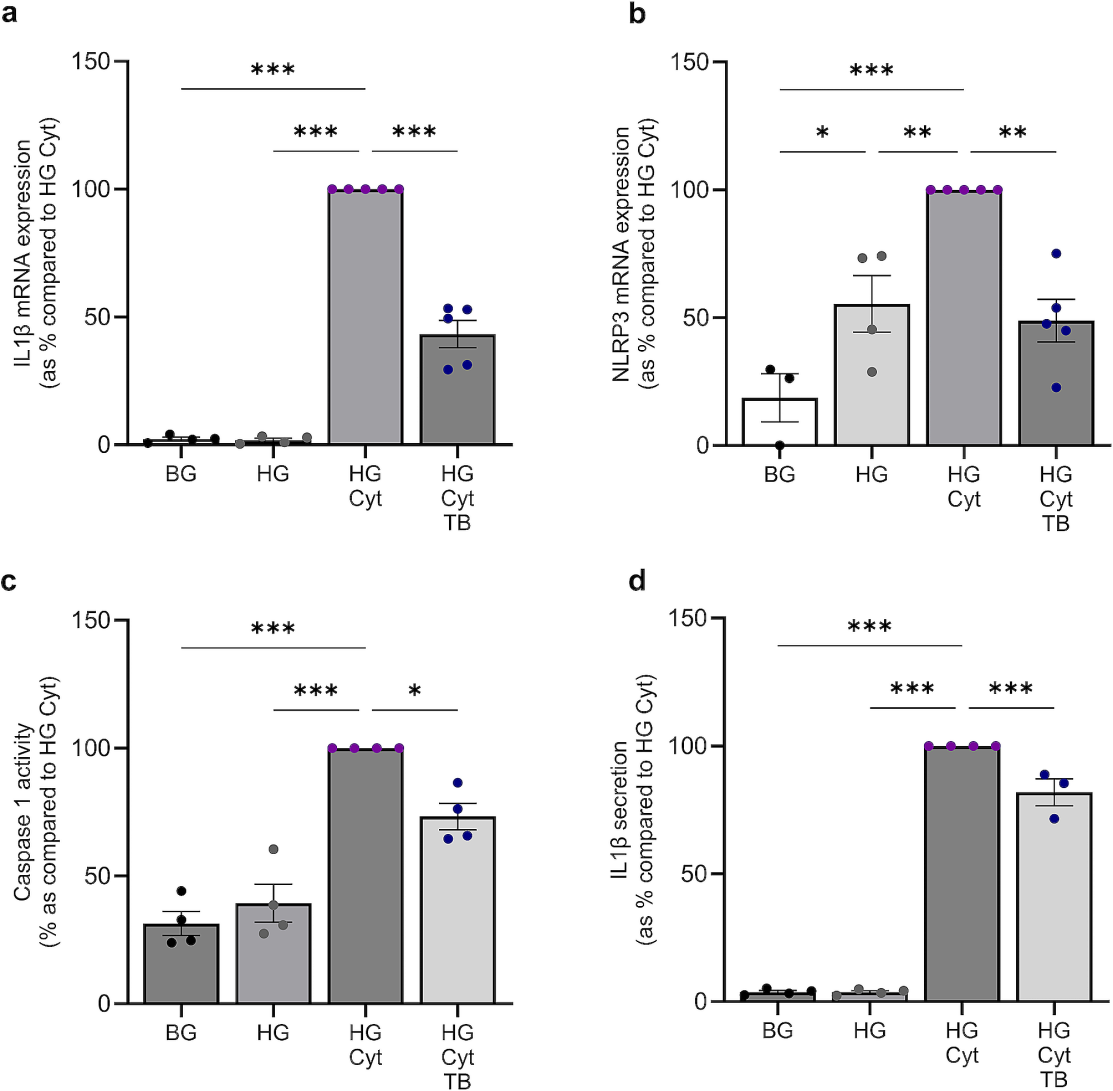
Tonabersat negates high glucose and cytokine-evoked increases in NLRP3 inflammasome priming and activation in human RPTECs. Human RPTECs were treated with 5mM/L basal glucose (BG) or 25mM/L high glucose (HG) +/- IL1β (10ng/mL) and TNFα (10ng/mL; Cyt) +/- Tonabersat (TB; 50µM) for 48 h. RT-qPCR evaluated changes in mRNA expression of IL1β **(a)** and NLRP3 **(b)**, normalised against GAPDH. Use of a caspase glo-1 assay assessed **(c)** caspase 1 activity whilst an ELISA assessed changes in IL1β secretion **(d)**. Date representative of *n =* 3–6 separate experiments. Significance is displayed as *P < 0.05, **P < 0.01, ***P < 0.001

Quantitative RT-PCR determined that Tonabersat partially negates the glucose and cytokine-mediated increase in inflammasome priming, as evidenced by a 57 ± 5.3% decrease in IL1β (*P <* 0.001; Fig. 3a) and a 51 ± 8.4% decrease in NLRP3 (*P <* 0.01; Fig. 3b) mRNA expression. The gold standard when measuring NLRP3 inflammasome activation is assessment of either caspase 1 activity or cleavage of IL1ß into its mature form. In support of our hypothesis, Tonabersat reduced caspase 1 activity (27 ± 5.1%; *P <* 0.05; Fig. 3c) and IL1β secretion (18 ± 5.3%; *P <* 0.001; Fig. 3d) following a high glucose and cytokine-mediated increase (69%; *P <* 0.001 and 96.3%; *P <* 0.001 respectively), suggesting that aberrant Cx43 hemichannel activity is upstream of both NLRP3 inflammasome priming and activation.

### Priming and activation of the NLRP3 inflammasome increases Cx43 expression and hemichannel activity

Findings by Alonso et al., suggest that transcription factor NFκB can bind to the Cx43 promoter, increasing Cx43 transcription [64, 65]. Furthermore, studies in retinal pigment epithelial cells determined that NLRP3 priming is linked to increased Cx43 expression, an event dependent upon increased NFκB activation [64, 65]. Having shown that aberrant Cx43 hemichannel activity primes and activates the NLRP3 inflammasome in RPTECs, we assessed if blocking NFκB in the presence of high glucose and cytokines had a direct effect on Cx43 expression and if this translated into a change in hemichannel function as evidenced by dye uptake and ATP release.

**Fig. 4:**
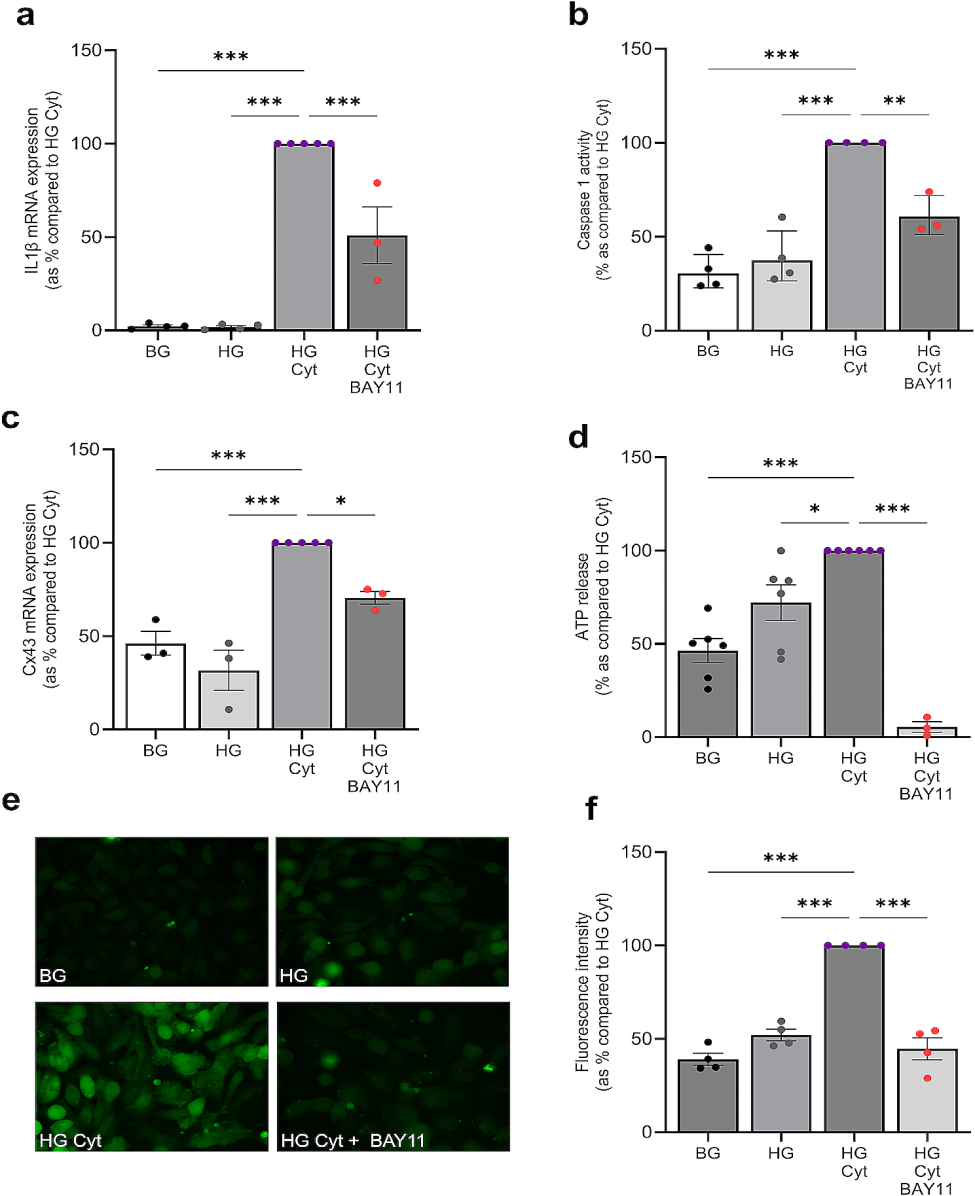
Blocking NFκB-mediated priming and activation of the NLRP3 inflammasome parallels a reduction in Cx43 expression and hemichannel activity. Human RPTECs were treated with 5mM/L basal glucose (BG) or 25mM/L high glucose (HG) +/- IL1β (10ng/mL) and TNFα (10ng/mL; Cyt) +/- BAY11 7082 (5µM) for 48 h. RT-qPCR evaluated changes in IL1β **(a)** mRNA expression, caspase 1 **(b)** activity and Cx43 **(c)** mRNA expression in response to inhibition of NFκB, when normalised against GAPDH. An ATPlite luminescence assay measured cellular release of ATP into the supernatant **(d)**. Carboxyfluorescein dye uptake studies determined changes in hemichannel number at the cell surface **(e)**, quantified using Fiji software **(f)**. Data representative of *n =* 3–6 separate experiments. Significance is displayed as *P < 0.05, **P < 0.01 ***P < 0.001

Pre-incubation of glucose and cytokine-treated cells with the NFκB inhibitor BAY11 7082 (BAY11) decreased IL1β mRNA expression (a measure of NLRP3 priming) by 49 ± 15% (*P <* 0.001; Fig. 4a) and caspase 1 activity (a measure of NLRP3 activation) by 39 ± 6.3% (*P <* 0.01; Fig. 4b) as compared to cells stimulated at high glucose with cytokines alone. Similarly, inhibition of NFκB decreased Cx43 transcription by 29 ± 3.4% (*P <* 0.05; Fig. 4c), reducing ATP release by 94.6 ± 2.8; (*P <* 0.001; Fig. 4d) and hemichannel-mediated dye uptake to 45 ± 5.9%; *P <* 0.001; Fig. 4e&f) as compared to cells treated with high glucose and cytokines alone.

The NLRP3 inflammasome mediates the innate immune inflammatory response through increased secretion of a large number of inflammatory mediators including IL1β, IL6 and TNFα [66]. With high glucose and inflammation having been shown to trigger signalling pathways that increase Cx43 hemichannel opening [37, 59, 60, 67–69], thereby exacerbating inflammation, we determined if blocking caspase 1 activity feeds forward to prevent inflammasome activation. Moreover, we investigated if this consequently reduced Cx43 expression and hemichannel-mediated dye uptake in response to diminished inflammation.

**Fig. 5:**
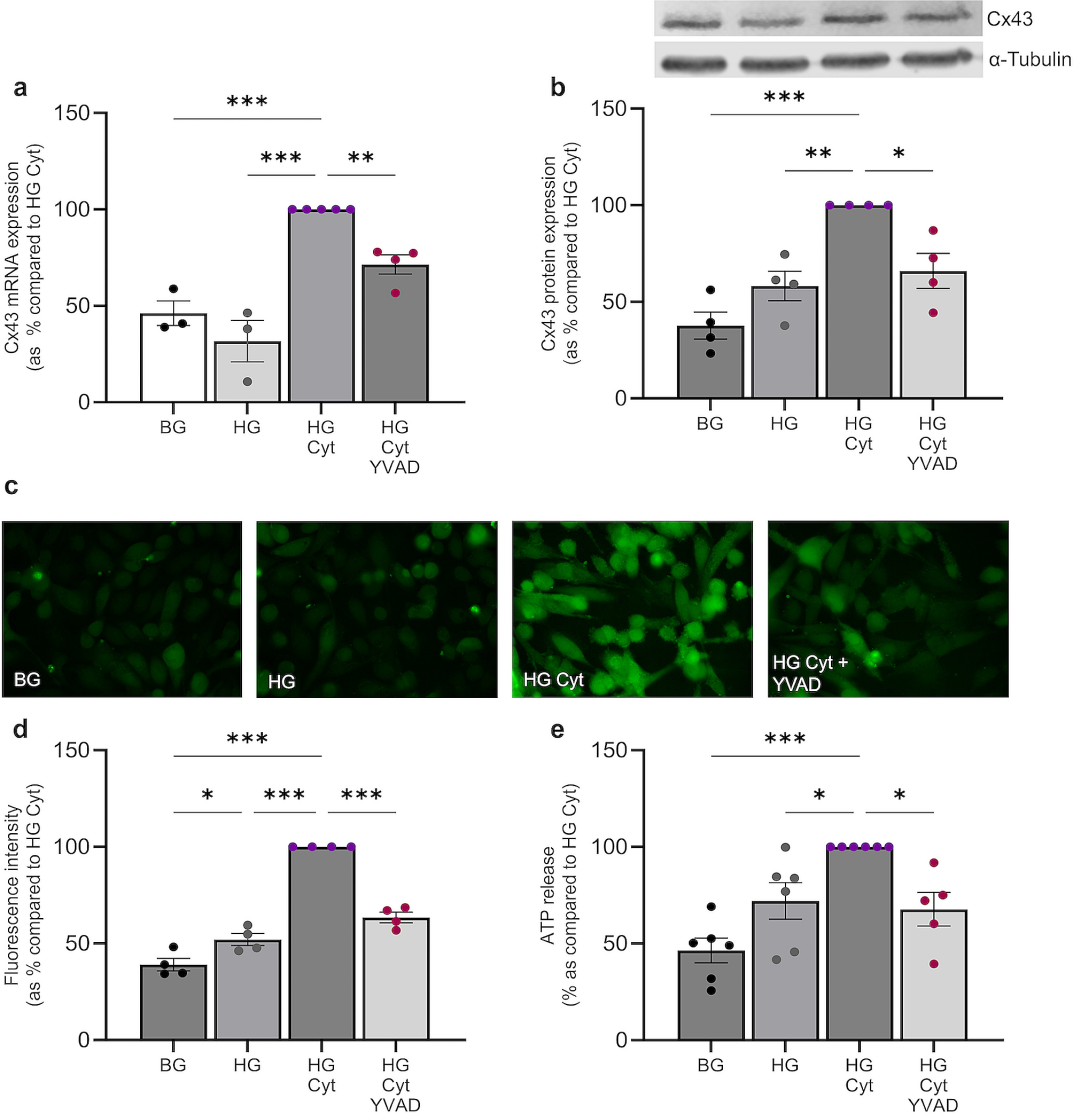
Blocking caspase 1 activity reduces pro-inflammatory cytokine-induced increases in Cx43 expression and hemichannel activity. Human RPTECs were treated with 5mM/L basal glucose (BG) or 25mM/L high glucose (HG) +/- IL1β (10ng/mL) and TNFα (10ng/mL; Cyt) +/- YVAD CMK (10µM) for 48 h. RT-qPCR evaluated changes in Cx43 mRNA expression normalised against GAPDH **(a)** whilst western blotting **(b)** assessed altered Cx43 protein expression. Results were normalised against expression of α-Tubulin as a loading control. Carboxyfluorescein dye uptake studies determined change in hemichannel number at the cell surface **(c)** which was quantified using Fiji software **(d)**. An ATPlite luminescence assay measured cellular release of ATP into the supernatant **(e)**. Data representative of *n =* 3–6 separate experiments. Significance is displayed as *P < 0.05, **P < 0.01, ***P < 0.001

Caspase 1 inhibitor YVAD CMK (10µM) decreased Cx43 mRNA (Fig. 5a) and protein expression (Fig. 5b) by 29 ± 5.0% (*P <* 0.01) and 34 ± 9.1% (*P <* 0.05), respectively. These findings are paralleled by a decrease in carboxyfluorescein dye uptake (37 ± 2.7%, *P <* 0.001; Fig. 5c and d) and hemichannel-mediated ATP release (32 ± 8.7%, *P <* 0.05; Fig. 5e) in high glucose and cytokine-treated RPTECs.

### Tonabersat blocks a high glucose and cytokine-induced increase in Cx43 expression, effects recapitulated in response to [Ca^2+^]_i_ chelation

**Fig. 6:**
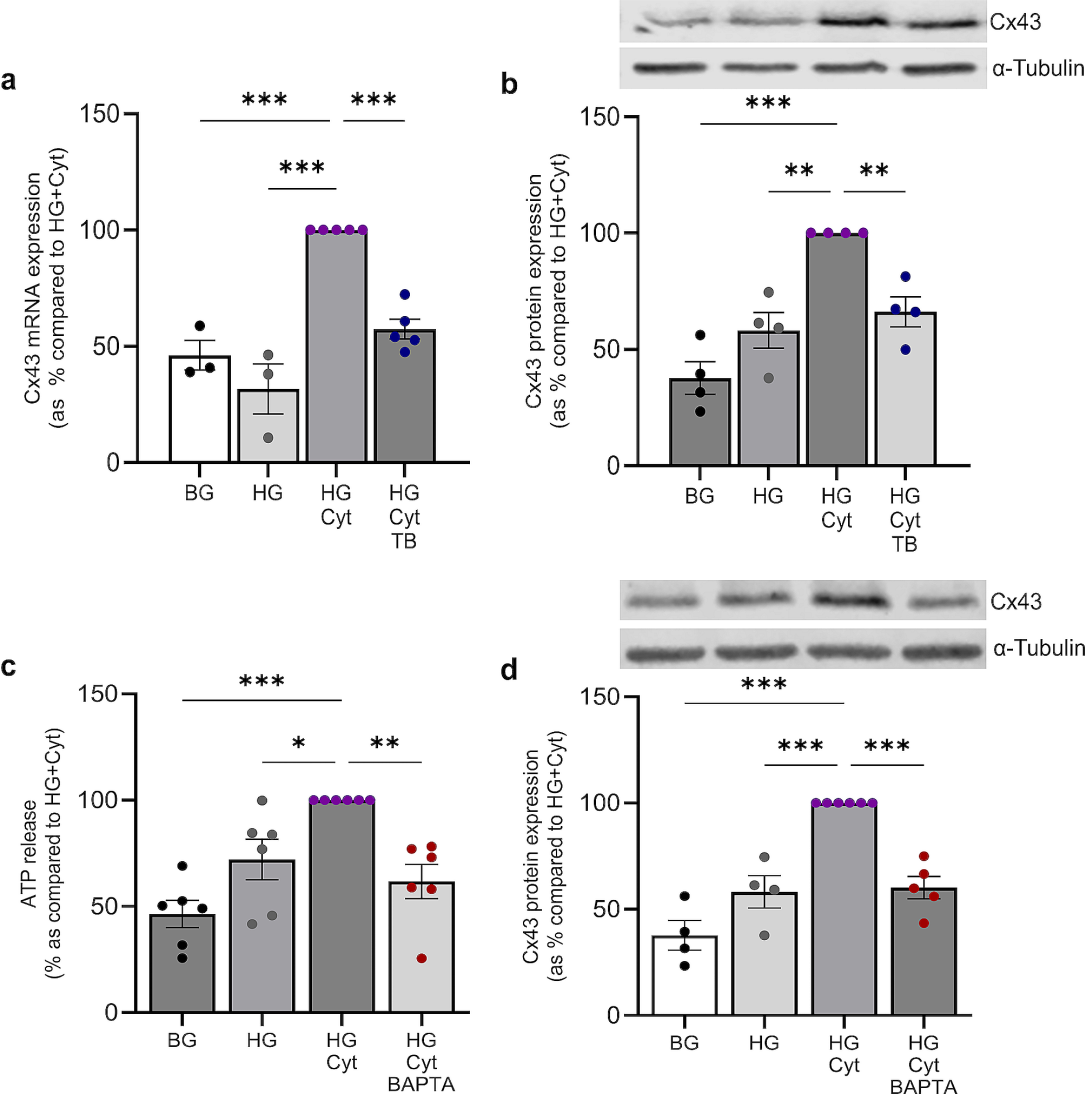
Tonabersat negates a glucose/proinflammatory cytokine-induced increase in Cx43 expression, associated with altered [Ca^2+^]_i_ gating. Human RPTECs were treated with 5mM/L basal glucose (BG) or 25mM/L high glucose (HG) +/- IL1β (10ng/mL) and TNFα (10ng/mL; Cyt) +/- Tonabersat (TB; 50µM) +/- BAPTA (5µM) for 48 h. RT-qPCR evaluated changes in Cx43 mRNA expression normalised against GAPDH **(a)** whilst western blotting **(b & d)** assessed altered Cx43 protein expression. Results were normalised against α-Tubulin as a loading control. An ATPlite luminescence assay measured cellular release of ATP into the supernatant **(c)**. Data is representative of *n =* 3–5 experiments. Significance is displayed as *P < 0.05, **P < 0.01 and ***P < 0.001

Since Tonabersat blocks NLRP3 activation (Fig. 3c&d) and inhibition of NLRP3 parallels a reduction in Cx43 expression (Fig. 5a&b), we further assessed if blocking Cx43 hemichannel activity has a direct inhibitory effect on Cx43 expression (a likely consequence of impaired downstream NLRP3 priming/activation). Primary human RPTECs pre-treated with Tonabersat prior to culturing with high glucose and cytokines exhibited a 43 ± 4.3% (*P <* 0.001) reduction in Cx43 mRNA (Fig. 6a) and 34 ± 6.4% (*P <* 0.01) reduction in Cx43 protein expression (Fig. 6b). These data suggest that Tonabersat breaks the cycle of events which lead to a reciprocal relationship between Cx43 hemichannel activity and NLRP3 inflammasome activity.

Increased intracellular calcium ([Ca^2+^]_i_) is a recognised gating stimulus of Cx43 hemichannel opening [34, 70]. Moreover, with assembly and activation of the inflammasome complex dependent upon ATP-P2 × 7 receptor-driven potassium/calcium efflux/influx, respectively [35, 71], this further supports the notion that inflammasome activation may worsen the state of inflammation through its ability to indirectly gate and open Cx43 hemichannels – thus releasing further DAMPS e.g., ATP. To test this, BAPTA was used to chelate [Ca^2+^]_i_ in the presence of high glucose and cytokines, mimicking the effects of hemichannel closure as induced by Tonabersat. BAPTA decreased both hemichannel-mediated ATP release (38 ± 8.1%; *P <* 0.01; Fig. 6c) and Cx43 protein expression (40 ± 5.3%; *P <* 0.001; Fig. 6d).

### Aberrant Cx43 hemichannel activity in RPTECs stimulates increased migration and expression of inflammatory chemokines in human-derived macrophages, effects blocked in part by Tonabersat

**Fig. 7:**
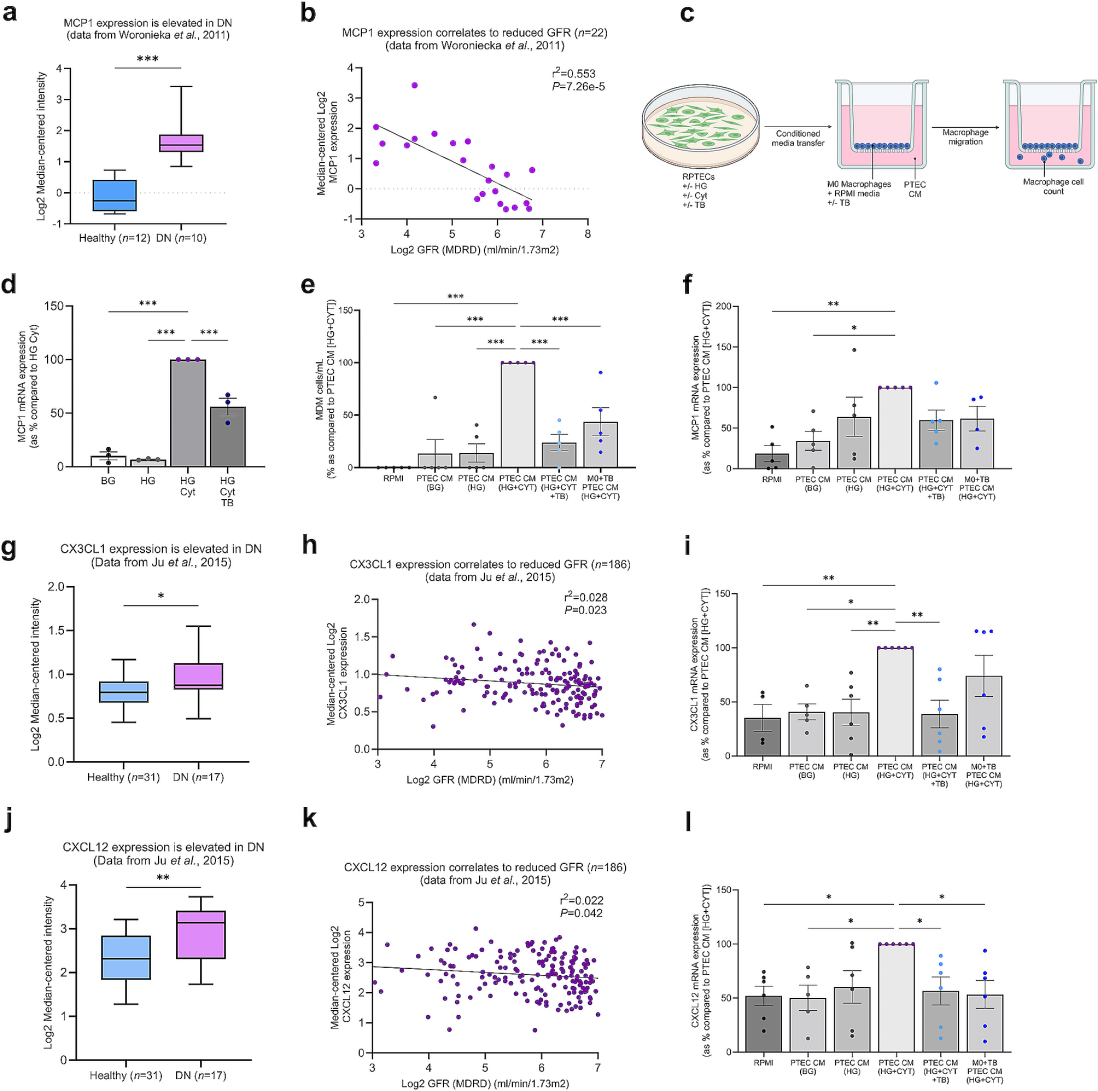
Tonabersat reduces tubular epithelial MCP1 expression and resultant paracrine-mediated macrophage migration. Transcriptomic analysis was performed on Nephroseq publicly available data [52, 54] comparing mRNA expression of MCP1 **(a)**, CX3CL1 **(g)** and CXCL12 **(j)** in the tubules of kidney biopsies from healthy donors and donors with diabetic nephropathy. The sample number (*n*) is specified where appropriate. Increased expression of each gene was further correlated to a declining GFR **(b, h&k** respectively**)**. An unpaired t-test with Welch’s correction analysis and simple linear regression were used for statistical analysis. Human RPTECs were treated with 5mM/L basal glucose (BG) or 25mM/L high glucose (HG) +/- IL1β (10ng/mL) and TNFα (10ng/mL; Cyt) +/- Tonabersat (TB; 50µM) for 48 h. RT-qPCR evaluated changes in MCP1 mRNA expression normalised against GAPDH **(d)**. Conditioned media (CM) generated from treated RPTECs +/- Tonabersat (TB; 50µM) **(c)** was applied to healthy human monocyte-derived macrophages (MDM) +/- Tonabersat (TB; 50µM) cultured in transwell plate inserts. **(e)** The number of MDMs migrating into the well containing RPTEC conditioned media was calculated. To support these observations, RT-qPCR evaluated changes in MCP1 **(f)**, CX3CL1 **(i)** and CXCL12 **(l)** mRNA expression normalised against GAPDH. Data representative of *n =* 3–6 experiments. Significance is displayed as *P < 0.05, ** *P* < 0.01 and ***P < 0.001

Having demonstrated that activity of Cx43 hemichannels and the NLRP3 inflammasome are reciprocally linked, we next delineated some of the downstream effects of this relationship. Recent findings by Roger et al., illustrate that tubule specific Cx43^−/−^ and Peptide 5 treatment reduces a UUO-mediated increase in macrophage infiltration in mice models [43]. Macrophages are the main immune cell involved in driving progression of DN, and a major source of pro-inflammatory cytokines and chemokines. Chemokine MCP1 is released from tubule cells involved in macrophage recruitment, a potential mechanism through which Cx43 contributes to upregulated accumulation. Transcriptomic analysis shows that MCP1 is upregulated in renal biopsy samples from people with DN as compared to healthy controls (*P <* 0.001; Fig. 7a), events that positively correlate with declining GFR (*P <* 0.001; Fig. 7b). No significant correlation with proteinuria was observed. In vitro, Tonabersat reduced a glucose and cytokine-mediated increase of MCP1 mRNA in human RPTECs by 46 ± 7.8% (*P <* 0.001; Fig. 7d), indicating a role for Cx43 hemichannel activity in tubule MCP1 expression.

To determine the effect of tubular ATP release and the associated increase in MCP1 expression on macrophage recruitment, healthy donor CD14 + monocytes were isolated, seeded and differentiated in transwell inserts. Conditioned media (CM) was generated from primary RPTECs cultured in either basal or high glucose, and high glucose + cytokines in the presence/absence of Tonabersat (Fig. 7c). This conditioned media was then used as a chemoattractant and added into the bottom chamber to form a chemotactic gradient (Fig. 7c). Macrophages sense the chemotactic gradient and migrate through the pores of the transwell membrane. The number of macrophages migrating towards RPTEC conditioned media was calculated as a measure of altered MDM motility/migration (Fig. 7e).

The number of migrating macrophages increased significantly when conditioned media from high glucose and cytokine treated RPTECs was used as a chemoattractant (*P <* 0.001; Fig. 7e), as compared to macrophage culture media alone or conditioned media from untreated or high glucose RPTECs. Importantly, migration was reduced in macrophages when exposed to conditioned media from Tonabersat-treated RPTECs (76 ± 7.6%; *P <* 0.001) and in macrophages that were pre-treated with Tonabersat prior to addition of conditioned media from high glucose and cytokine treated RPTECs (56 ± 13%; *P <* 0.001).

With macrophages being an important source of MCP1 which can further contribute to the recruitment of immune cells, we observed that conditioned media from high glucose and cytokine treated RPTECs triggered an increase in macrophage MCP1 mRNA expression of 77% (*P* < 0.01) and 57% (*P <* 0.05) as compared to culture media alone or conditioned media from untreated RPTECs respectively (Fig. 7f). However this effect, whilst stimulated by our PTEC secretome (high glucose and cytokine-treated conditioned media), was not significantly reduced in the presence of conditioned media in which RPTECs had been pretreated with Tonabersat, or in macrophages pre-treated with Tonabersat prior to addition of RPTEC conditioned media (Fig. 7f).

In addition to MCP1, we investigated a role for chemokines CX3CL1 (Fig. 7g-i) and CXCL12 (Fig. 7j-l) in DN, and examined the role that aberrant RPTEC Cx43 hemichannel-mediated ATP release may play in regulating expression of these genes in macrophages. Studies highlight that CX3CL1 and CXCL12 promote macrophage polarisation [72], migration [73] and monocyte-to-macrophage differentiation, respectively [74, 75]. Their role in kidney disease is not widely reported. Using renal transcriptomic data available on the Nephroseq repository we determined that expression of CX3CL1 (*P* < 0.05; Fig. 7g) and CXCL12 (*P* < 0.01; Fig. 7j) is increased in diseased kidneys (*n* = 31) as compared to healthy donor controls (*n* = 17). Subsequent analysis determined that increased CX3CL1 (*P* < 0.05; Fig. 7h) and CXCL12 (*P* < 0.05; Fig. 7k) expression positively correlates with declining GFR in disease.

Transfer of high glucose and cytokine treated RPTEC conditioned media evoked a significant increase in CX3CL1 as compared to macrophage culture media alone (64.8%; *P <* 0.01), or conditioned media from untreated (59.3%; *P <* 0.05) and high glucose (59.5%; *P <* 0.01) treated RPTECS (Fig. 7i). Interestingly, CX3CL1 expression was significantly reduced by 61 ± 12.8% (*P <* 0.01; Fig. 7i), in the presence of conditioned media in which RPTECs had been pre-treated with Tonabersat, whilst inhibition of macrophage hemichannels prior to application of RPTEC conditioned media had no effect. Contrary to CX3CL1, the high glucose and cytokine-treated RPTEC conditioned media induced an increase in CXCL12 expression as compared to macrophage culture media alone (48%; *P <* 0.05), and conditioned media from untreated (50%; *P <* 0.05) RPTECS alone (Fig. 7l) was significantly blunted when Cx43 hemichannels were blocked in both RPTECs (44%±12.9%; *P* < 0.05) and macrophages (47%±12.9%; *P* < 0.05; Fig. 7l), thus suggesting a role for Cx43 hemichannels across both cell types in orchestrating this response.

### Aberrant Cx43 tubule hemichannel activity produces an RPTEC secretome that drives macrophage polarisation

**Fig. 8:**
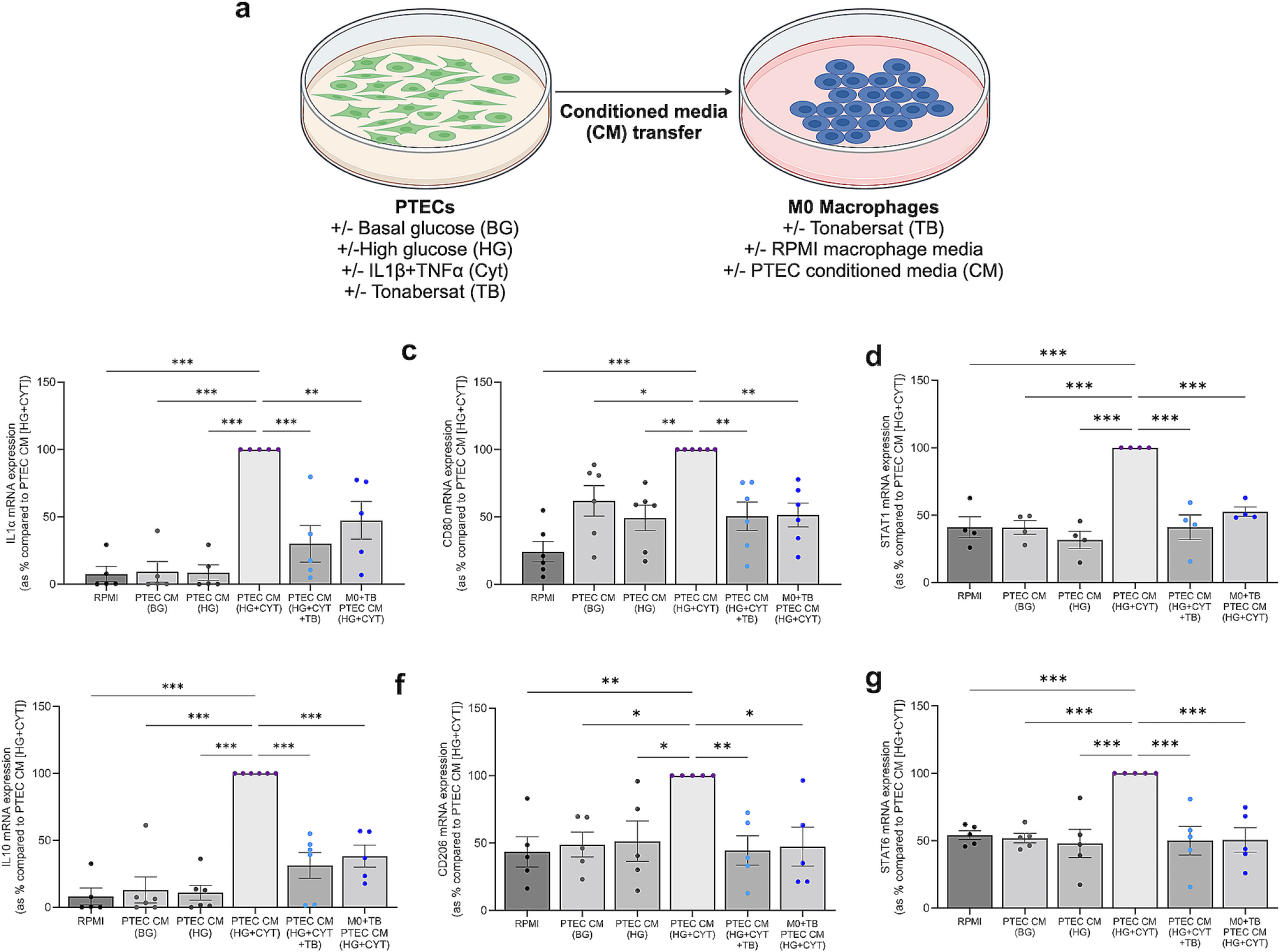
Blocking Cx43 hemichannels in tubule cells mitigates glucose and inflammatory cytokine-induced paracrine-mediated changes in macrophage polarisation and inflammation. Conditioned media was generated from human RPTECs treated with 5mM/L basal glucose (BG) or 25mM/L high glucose (HG) cultured in IL1β (10ng/mL) and TNFα (10ng/mL; Cyt) +/- Tonabersat (TB; 50µM). This was then applied to healthy human monocyte derived macrophages (MDM) +/- Tonabersat (TB; 50µM) **(a)**. RT-qPCR evaluated changes in expression of both M1 macrophage markers IL1α **(b)**, CD80 **(c)** and STAT1 **(d)** and M2 markers IL10 **(e)**, CD206 **(f)** and STAT6 **(g)**. Data representative of *n =* 4–6. Significance is displayed as *P < 0.05, **P < 0.01, ***P < 0.001

In addition to increased motility, tubule-derived factors have been previously reported to preferentiality polarise macrophages to a pro-inflammatory M1 phenotype [76–78], often associated with increased inflammation and early damage to the kidney tubules [79]. Conditioned media transfer using high glucose and cytokine-treated RPTEC conditioned media (Fig. 8a), increased the expression of pro-inflammatory marker IL1α (92.5; *P <* 0.001; Fig. 8b), M1 marker CD80 (76%; *P <* 0.001; Fig. 8c), and signal transducer and activator of transcription- (STAT)1 (59%; *P <* 0.001; Fig. 8d) in human primary MDMs as compared to macrophage culture media alone. These changes were significantly reduced by 70 ± 14% (*P <* 0.001), 49 ± 10% (*P <* 0.01), and 59 ± 9.1% (*P <* 0.001), respectively when macrophages were cultured in the presence of RPTEC-conditioned media generated from RPTECs in which Cx43 hemichannels had been blocked. Furthermore, when Tonabersat is applied to macrophages directly prior to addition of conditioned media, the expression of IL1α (53 ± 14%; *P <* 0.01; Fig. 8b), CD80 (49 ± 8.9%; *P <* 0.01; Fig. 8c) and STAT1 (47 ± 3.5%; *P <* 0.001; Fig. 8d), was reduced as compared to the effect of conditioned media alone.

Although M2 macrophages are typically considered anti-inflammatory and reparative in nature, prolonged activation in the face of damaging stimuli contributes to tubulointerstitial fibrosis [80]. To determine a role for Cx43 hemichannel activity in regulating M2 marker expression, the effect of high glucose and cytokine treated RPTEC conditioned media on expression of M2 markers, IL10, CD206 and STAT6 in macrophages was assessed. High glucose and cytokine-induced conditioned media increased expression of IL10, CD206 and STAT6 by 91.8% (*P* < 0.001; Fig. 8e), 57% (*P* < 0.01; Fig. 8f), and 46% (*P* < 0.001; Fig. 8g), respectively as compared to macrophage culture media alone. These changes were significantly reduced by 69 ± 9.6% (*P <* 0.001), 56 ± 11% (*P <* 0.01), and 50 ± 11% (*P <* 0.001), respectively, when macrophages were cultured in the presence of RPTEC conditioned media generated from RPTECs in which Cx43 hemichannels had been blocked. This effect was recapitulated when macrophages were preincubated with Tonabersat for IL10 (62 ± 8.1%; *P <* 0.001; Fig. 8e), CD206 (53 ± 14%; *P* < 0.05; Fig. 8f) and STAT6 (49 ± 9.1%; *P <* 0.001; Fig. 8g). Collectively the data suggest a role for Cx43 hemichannel-mediated signalling in the polarisation of M1 and M2 macrophages.

### Aberrant Cx43 tubule hemichannel activity produces a RPTEC secretome that promotes NLRP3 inflammasome activation in macrophages

The relationship between the NLRP3 inflammasome and its link to Cx43 hemichannel activity is reported here in RPTECs cultured under conditions of high glucose in the presence of inflammatory cytokines. Furthermore, inhibition of Cx43 hemichannel activity in macrophages significantly reduces expression of macrophage inflammatory markers as induced by the conditioned media from high glucose and cytokine treated RPTECs.

**Fig. 9:**
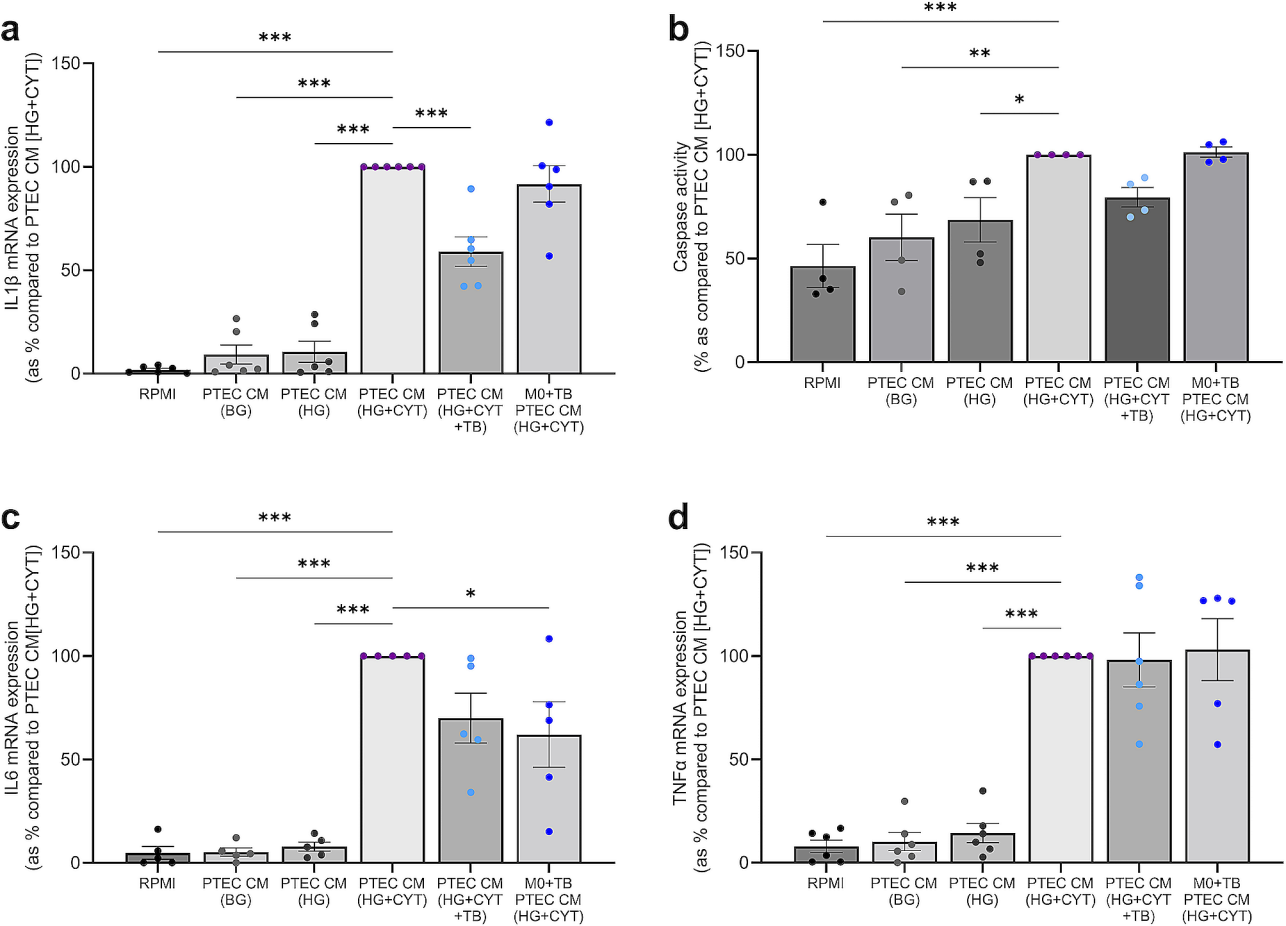
Blocking Cx43 hemichannels in tubule cells and macrophages mitigates glucose and inflammatory cytokine-induced paracrine-mediated changes in macrophage inflammation. Conditioned media was generated from human RPTECs treated with 5mM/L basal glucose (BG) or 25mM/L high glucose (HG) cultured in IL1β (10ng/mL) and TNFα (10ng/mL; Cyt) +/- Tonabersat (TB; 50µM). This was then applied to healthy human monocyte-derived macrophages (MDMs) +/- Tonabersat (TB; 50µM). Changes in mRNA expression of IL1β **(a)**, caspase 1 activity **(b)** IL6 mRNA **(c)** and TNFα mRNA expression **(d) were assessed**. Data representative of *n =* 5–6. Significance is displayed as *P < 0.05, **P < 0.01, ***P < 0.001

We assessed if Cx43 hemichannel activity in both cell types contributes to NLRP3 priming and activation in macrophages and whether this was a consequence of epithelial-to-macrophage and/or macrophage-to-macrophage crosstalk. A measure of inflammasome priming, expression of IL1β mRNA increased in macrophages cultured in high glucose and cytokine treated RPTEC media (98.1%; *P <* 0.001; Fig. 9a) as compared to macrophage media, a response significantly reduced when cultured in RPTEC conditioned media which had been generated in the presence of Tonabersat (41 ± 7.1%; *P <* 0.001). Furthermore, high glucose and cytokine treated RPTEC conditioned media triggered an increase in caspase 1 macrophage activity (54 ± 10%; *P <* 0.001; Fig. 9b) as compared to macrophage culture media, however this effect was not abolished when Cx43 hemichannels were blocked in either cell type.

As downstream products of the NLRP3 inflammasome, RPTEC-CM evoked a significant increase in mRNA expression of IL6 and TNFα by 95.2% (*P <* 0.001; Fig. 9c) and 92.1% (*P <* 0.001; Fig. 9d) respectively in macrophages. However, whilst treating RPTECs with Tonabersat did not reduce the effect of the RPTEC-CM on macrophage expression of IL6 or TNFα, in this instance, macrophages pre-treated with Tonabersat prior to treatment with conditioned media responded favourably, exhibiting a significant reduction in IL6 expression by 38 ± 16% (*P <* 0.05; Fig. 9c).

These data suggest that the RPTEC secretome contains secretory factors which (released downstream of aberrant Cx43 hemichannel activity) evoke increased paracrine-mediated priming of the NLRP3 inflammasome and inflammation in human MDMs.

## Discussion

Like other chronic age-related diseases, individuals with kidney disease have an accelerated ageing phenotype caused by convergence of fundamental mechanisms that underlie age-related tissue dysfunction, including chronic “sterile” (absence of infection) NLRP3-induced inflammation [81]. Such events are compounded by the presence of diabetes. With several studies in different tissue types linking altered connexin activity to inflammation [43, 82–86], senescence [87, 88], and fibrosis [89–91] across multiple disease states, evidence suggests that stabilising hemichannel and/or gap junction-mediated communication, may be a viable approach to prevent the onset of tissue damage that develops with secondary complications of diabetes, including retinopathy. Links between connexins and NLRP3-induced inflammation in DN remain to be fully identified.

In the current study, we generated an in vitro model of human DN and describe the ability of Tonabersat to block a perpetual feedforward cycle between Cx43 hemichannel activity and NLRP3 inflammasome priming/activation. Moreover, with tubulointerstitial inflammation contributed to by multiple cell types [92–95], we evidence a role for Cx43 hemichannels in heterotypic cell-to-cell signalling, a role attributable to aberrant hemichannel-induced tubule-derived secretory factors acting via paracrine-mediated signalling on primary human monocyte-derived macrophages. These observations may explain reduced macrophage infiltration in our previously reported tubule site-directed Cx43^−/−^ UUO mouse model of advanced kidney disease [43].

This is the first study that identifies a protective role for Tonabersat in blocking sterile NLRP3-induced inflammation in primary kidney and immune cells. Analysis of human transcriptomic datasets (Fig. 2) determined an increase in markers of inflammasome priming and activation in biopsy material isolated from individuals with DN, changes paralleled by a decline in renal function. Due to the critical role the NLRP3 inflammasome plays in the pathogenesis of chronic inflammatory diseases, there have been numerous attempts to target its activity across multiple diseases [96]. However, whilst several different therapeutic targets have been considered, experimental compounds that target the NLRP3 inflammasome exclusively are yet to reach their endpoint, with OLT1177 (DAPANSUTRILE^®^; Clinical Trials Identifier: NCT04540120), and DFV890 (IFM-2427; Clinical Trials Identifier: NCT04382053) each still in clinical trial. An absence of food and drug administration (FDA) approved drugs may reflect potential issues surrounding inhibition of both sterile inflammation and the innate immune response, where activation of the NLRP3 inflammasome plays a pivotal role in defence against pathogens [97–99]. To avoid disrupting the innate immune response, compounds that work upstream of the inflammasome could be more effective.

Studies in diabetic retinopathy indicate that Cx43 hemichannels lie upstream of inflammasome activity, with aberrant ATP release activating the P2 × 7 receptor, NLRP3 assembly and resultant caspase 1-mediated cleavage of IL1ß and IL18 [37]. With numerous studies demonstrating a causal role for Cx43 expression and activity in NLRP3 activation [32, 43, 60], connexin hemichannel blockers (Clinical Trials Identifier: NCT05727891) and gap junction modifiers (Clinical Trials Identifier: NCT01977755) [100] are now in clinical trial. Given the inextricable link between retinopathy and nephropathy [101, 102], an amendment to assess biomarkers of renal function in patients with diabetic macular oedema treated with Tonabersat has recently been announced (Clinical Trials Identifier: NCT05727891).

Findings by Xu et al., showed that a progressive increase in Cx43 expression correlates with the severity of injury in the mouse UUO model of advanced kidney inflammation and fibrosis [50]. In support of these observations, our earlier studies using a UUO-Cx43^−/−^ tubule-specific knockout [43] and UUO-Cx43^+/−^ global mouse [103] demonstrated that either a reduction in whole animal expression or site directed tubule knockout of Cx43 has beneficial effects on the expression of disease markers. Data from the UUO-Cx43^−/−^ tubule knockout model suggests a role for Cx43 in mediating inflammasome activation, with Cx43 knockout reducing gene expression of markers of inflammasome priming and activation [43]. Additionally, in a model of acute renal injury induced by lipopolysaccharide (LPS), heterozygous Cx43^+/−^ mice had significantly reduced inflammasome activation as compared with wild-type Cx43^+/+^, as demonstrated by lower levels of blood IL1β and renal expression of NLRP3, culminating in reduced blood urea nitrogen (BUN), proteinuria and renal pathological changes [51]. In the current study, we demonstrate that Tonabersat, an orally bioavailable Cx43 hemichannel blocker (NCT05727891), which has previously demonstrated a good safety profile in phase II clinical trials for migraine prophylaxis [104], reduces Cx43 hemichannel-mediated priming and activation of the NLRP3 inflammasome in RPTECs in response to high glucose and inflammatory cytokines. These observations are paralleled by a reduction in downstream inflammatory mediators (e.g., IL1β). It is possible that ATP released from hemichannels activates cell surface receptors to induce inflammasome priming [105] whilst simultaneously triggering P2 × 7-mediated inflammasome activation [71]. On the other hand, increased release of inflammatory cytokines (including IL1β and TNFα) following activation, may also drive NFκB-mediated priming via interleukin 1 receptor (IL1-R) and tumour necrosis factor receptor (TNFR) stimulation. Moreover, inflammation is a recognised gating stimulus of hemichannels, further contributing to aberrant hemichannel-mediated ATP release under diseased conditions [37, 68, 106]. Based on these findings and having ascertained that tubular Cx43 hemichannel activity drives NLRP3 priming and activation in an in vitro model of DN, we sought to identify a reciprocal link between inflammasome activation and the expression and function of Cx43. Data suggest that inflammasome priming and activation both contribute to increased Cx43 expression and hemichannel activity, indicating a vicious cycle of inflammation driven by the reciprocal relationship between hemichannel and inflammasome activity.

In other models of disease, NFκB binds to the Cx43 promoter upregulating its expression [64], a mechanism by which NLRP3 priming might increase Cx43 expression and a suggestion supported by our data showing that blocking NFκB activation reduces Cx43 expression in primary proximal tubule cells. Potential mechanisms by which inflammasome activation contributes to Cx43 hemichannel activity include the subsequent release of pro-inflammatory cytokines, e.g., IL1β and TNFα, which lead to increased Cx43 expression in the presence of high glucose. Moreover, a P2 × 7-mediated influx of Ca^2+^ ions could result in the opening of Cx43 hemichannels [70]. Data using Tonabersat shows that targeting upstream of the inflammasome, thus leaving it available for microbial defence, prevents formation of a detrimental feedforward loop and reduces downstream inflammation.

Activation of the inflammasome affects kidney structure [107, 108], and leads to fibrosis [109, 110] and the recruitment of immune cells, primarily macrophages [111, 112]. Tubular deletion of Cx43 successfully blocks these downstream effects in vivo, reducing macrophage infiltration and protecting against renal injury [43]. Xu et al., demonstrated that increased tubular Cx43 expression in UUO mice is paralleled by increased macrophage infiltration. However, without use of a specific hemichannel blocker, the precise mechanisms that underpin the effect of proximal tubule Cx43 hemichannel activity on the recruitment and activation of macrophages has yet to be identified [50]. Expression of MCP1, a chemokine involved in macrophage recruitment is increased in the kidneys of people with DN [113] and is similarly elevated in our high glucose and cytokine-treated RPTECs. Blocked by preincubation with Tonabersat, our data suggest that tubular Cx43 hemichannel activity may drive excessive recruitment of macrophages in DN through increased MCP1 secretion. Research by Xu et al., showed that whole kidney mRNA expression of chemo-attractants GM-CSF and MCP1 were increased in mice following unilateral ischemia/reperfusion injury, a model of maladaptive kidney repair after transient kidney injury, which positively correlated to progressive macrophage accumulation and collagen-I expression [114]. Furthermore, using co-cultured naïve bone marrow derived-macrophages with serum-starved mouse proximal tubular cells, they established that increased macrophage expression of MCP1 is dependent on tubule expression of GM-CSF [114], highlighting a role for proximal tubule derived factors in macrophage recruitment.

In the current study, we generated conditioned media from high glucose and cytokine treated RPTECs and applied both indirectly to healthy donor human monocyte-derived macrophages cultured on transwells, and directly via conditioned media transfer. In doing so, we assessed the effect of paracrine-mediated signalling on macrophage motility, polarity, NLRP3 inflammasome activity and inflammatory expression profile. Pre-incubation of cells with or without Tonabersat prior to generating conditioned media elucidated the contribution of Cx43 hemichannel activity when applied to RPTECs, macrophages or both. Data demonstrate that when RPTECs were preincubated with Tonabersat, increased conditioned media-induced macrophage migration, events important for infiltration into the tissue [115], were effectively blocked. Similarly, a reduction in mRNA expression of pro-inflammatory M1 (IL1α, CD80, STAT1) and resolving M2 (IL10, CD206 and STAT6) markers were observed, changes which paralleled dampened inflammasome priming (IL1β) and expression of pro-inflammatory markers (IL6 and TNFα). Macrophages represent one of the main sources of pro-inflammatory factors in the diabetic kidney [116], with prolonged M1 and M2 activation culminating in inflammation and fibrosis respectively. Our data identifies a potential beneficial mechanism through which hemichannel blockers may protect from the inflammatory burden of this disease. Furthermore, when Tonabersat was applied to macrophages prior to the addition of conditioned media, macrophage recruitment, inflammasome priming and expression of M1, M2 and inflammatory markers was also significantly reduced. These observations support a pathophysiological role for immune cell Cx43 hemichannel activity in contributing to the damage observed in the interstitium of the diabetic kidney and are observations are further corroborated by studies demonstrating that mouse peritoneal macrophages stimulated in combination with LPS and ATP show a Cx43-mediated increase in NLRP3 inflammasome activity, effects suppressed by Cx43 siRNA [51]. Taken together this data highlights potentially protective effects of Tonabersat in multiple cell types involved in the progression of kidney disease.

**Fig. 10:**
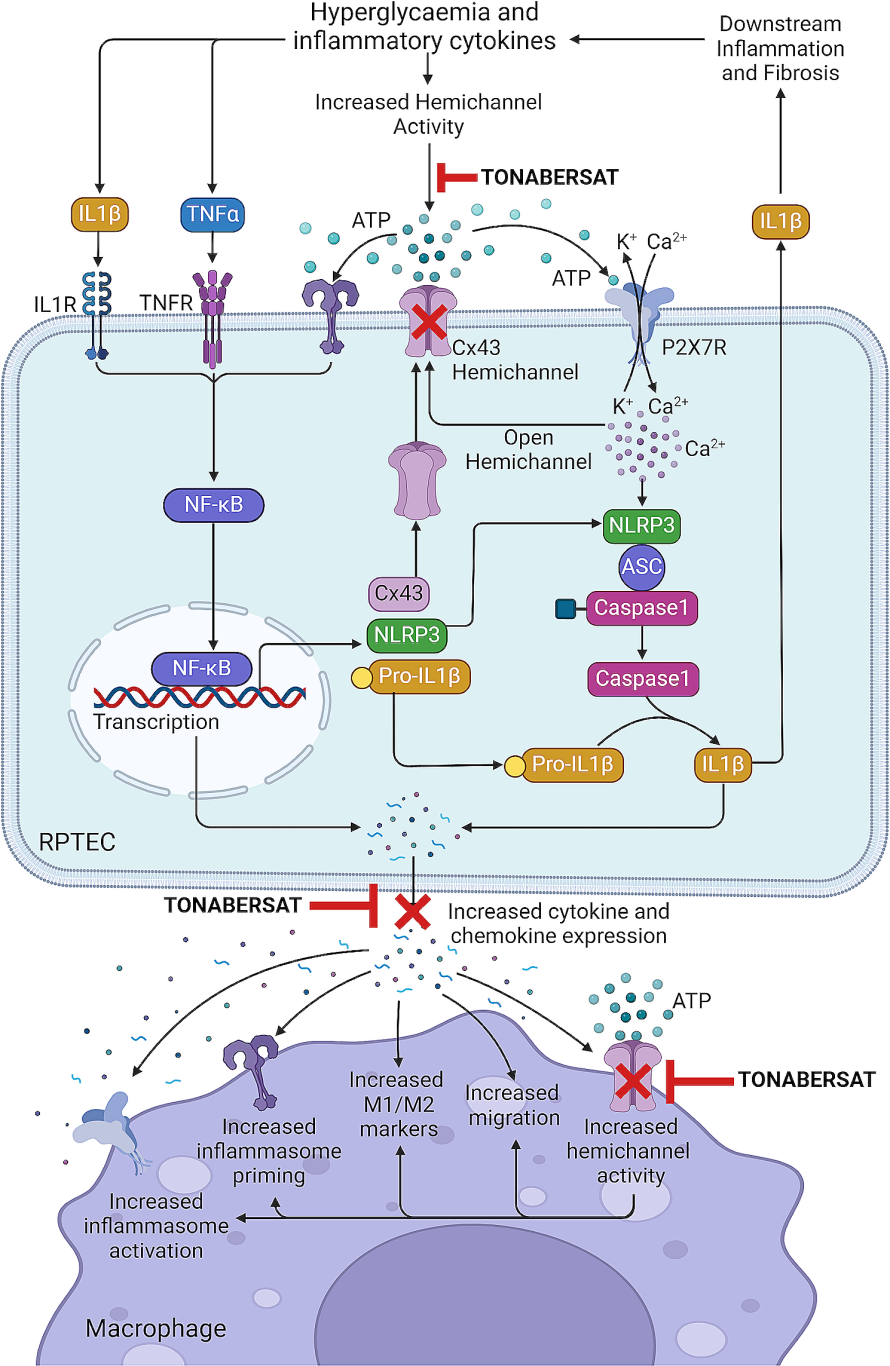
Role of Cx43 hemichannels in induction of injury in a model of diabetic nephropathy via NLRP3 inflammasome priming/activation and paracrine-mediated cell-cell communication. Under hyperglycaemic and pro-inflammatory conditions, proximal tubule epithelial cell Cx43 hemichannels open, releasing ATP. Known to activate purinergic P2 × 7 receptors, ATP leads to K^+^ efflux and Ca^2+^ influx, inducing NLRP3 inflammasome complex formation, caspase 1 activation, cleavage of pro-IL1β and release of mature IL1β into the extracellular space. This, in turn, drives signalling pathways culminating in downstream inflammation and fibrosis, including NLRP3 inflammasome priming via translocation of NFκB to the nucleus. Inflammasome priming and activation lead to increased Cx43 expression, elevated [Ca^2+^]_i_ and increased inflammation, all of which contribute to a further increase in Cx43 hemichannel activity, creating a feedforward loop. As a result of increased inflammatory cytokine and chemokine release, injured RPTECs increased migration, polarisation marker expression and inflammasome priming/activation in donor monocyte-derived macrophages. Hemichannel blocker Tonabersat reduces aberrant Cx43 hemichannel activity in RPTECs and macrophages to negate downstream changes induced by high glucose and inflammation. Created using Biorender.com

In summary, we provide evidence of beneficial effects of Tonabersat in blocking Cx43 hemichannel-mediated ATP release in an in vitro model of human DN, negating downstream NLRP3 priming/activation, macrophage polarisation/motility and tubule cell/macrophage expression of pro-inflammatory mediators. We show for the first time that inflammasome priming and activation drive increased hemichannel activity to perpetuate chronic inflammation in an in vitro model of DKD. In blocking Cx43 hemichannel-mediated inflammasome activation, Tonabersat may represent an effective adjunct therapy for slowing progression of tubular inflammation of the diabetic kidney (Fig. 10). In addition, Cx43 hemichannel blockers may offer polypharmacological benefits, suppressing common mechanisms that underpin inter-organ inflammatory damage in both the diabetic eye and kidney. Whilst our work unveils a potential therapeutic role for Tonabersat in suppression of key inflammatory pathways and paracrine mediated cell crosstalk, future studies are now required to determine the efficacy of Tonabersat or alternative Cx43 hemichannel blockers in models of DKD, notably those where full renal function can be assessed.

## Data Availability

No datasets were generated or analysed during the current study.

## Abbreviations

[Ca^2+^]_i_: Intracellular calcium concentration
ANOVA: Analysis of variance
ASC: Apoptosis-associated speck-like protein containing a C-terminal caspase recruitment domain
ATP: Adenosine triphosphate
BAPTA: AM − 1,2-bis(o-aminophenoxy)ethane-N, N,N′,N′-tetraacetic acid
BSS: Balanced salt solution
BUN: Blood urea nitrogen
C: Complementary
CASP1: Caspase 1
CD: Cluster of differentiation
CM: Conditioned media
CMT: Conditioned media transfer
Cx43: Connexin-43
Cyt: Cytokine
DAMP: Damage-associated molecular pattern
DN: Diabetic nephropathy
EGTA: Ethylene glycol-bis(β-aminoethyl ether)-N, N,N′,N′-tetraacetic acid
ELISA: Enzyme-linked immunosorbent assay
ESRD: End-stage renal disease
FCS: Foetal calf serum
FDA: Food and drug administration
GAPDH: Glyceraldehyde-3-phosphate dehydrogenase
GCSF: Granulocyte colony stimulating factor
GFR: Glomerular filtration rate
GLP: 1-Glucagon-like peptide 1
HEPES − 4: (2-hydroxyethyl)-1-piperazineethanesulfonic acid
ICAM1: Intercellular adhesion molecule-1
IL: Interleukin
IL1: R-Interleukin 1 receptor
LPS: Lipopolysaccharide
MCP1: Monocyte chemoattractant protein-1
M: CSF-Macrophage colony stimulating factor
MDMs: Monocyte-derived macrophages
NFκB: Nuclear Factor Kappa-B
NLRP3: NOD-like receptor protein 3 inflammasome
P2X7: Purinergic Receptor P2 × 7
PBMCs: Peripheral blood mononuclear cells
PBS: Phosphate buffered saline
RAS: Renin angiotensin system
RCF: Relative centrifugal force
RPE: Retinal pigment epithelial
RPTECs: Renal proximal tubule epithelial cells
RT: qPCR-Real-time quantitative polymerase chain reaction
SEM: Standard error of the mean
SGLT2: Sodium-glucose co-transporter-2
siRNA: Small interfering RNA
STAT: Signal transducer and activator of transcription
TB: Tonabersat
TNFR: Tumour necrosis factor receptor
TNFα: Tumour necrosis factor alpha
U: IRI-Unilateral ischemia/reperfusion injury
UUO: Unilateral ureteral obstruction
VEGF: Vascular endothelial growth factor
